# Distant Supervision with Transductive Learning for Adverse Drug Reaction Identification from Electronic Medical Records

**DOI:** 10.1155/2017/7575280

**Published:** 2017-09-26

**Authors:** Siriwon Taewijit, Thanaruk Theeramunkong, Mitsuru Ikeda

**Affiliations:** ^1^The School of Information, Communication and Computer Technologies, Sirindhorn International Institute of Technology, Thammasat University, Pathum Thani 12120, Thailand; ^2^The School of Knowledge Science, Japan Advanced Institute of Science and Technology, Nomi 923-1292, Japan

## Abstract

Information extraction and knowledge discovery regarding adverse drug reaction (ADR) from large-scale clinical texts are very useful and needy processes. Two major difficulties of this task are the lack of domain experts for labeling examples and intractable processing of unstructured clinical texts. Even though most previous works have been conducted on these issues by applying semisupervised learning for the former and a word-based approach for the latter, they face with complexity in an acquisition of initial labeled data and ignorance of structured sequence of natural language. In this study, we propose automatic data labeling by distant supervision where knowledge bases are exploited to assign an *entity-level* relation label for each drug-event pair in texts, and then, we use patterns for characterizing ADR relation. The multiple-instance learning with expectation-maximization method is employed to estimate model parameters. The method applies transductive learning to iteratively reassign a probability of unknown drug-event pair at the training time. By investigating experiments with 50,998 discharge summaries, we evaluate our method by varying large number of parameters, that is, pattern types, pattern-weighting models, and initial and iterative weightings of relations for unlabeled data. Based on evaluations, our proposed method outperforms the word-based feature for NB-EM (iEM), MILR, and TSVM with F1 score of 11.3%, 9.3%, and 6.5% improvement, respectively.

## 1. Introduction

Data-driven approach for knowledge extraction from electronic medical records (EMRs) has gained much attention in recent years. An EMR repository contains a collection of tacit knowledge [1] (e.g., professionals' experiences, know-how) and explicit knowledge (e.g., diagnosis procedure, patient information) in a digital form of structured and unstructured data. This EMR repository offers insight into significant healthcare problems: patient mortality prediction [2], patient risk identification [3, 4], drug-disease relation extraction [5], and drug-drug interaction prediction [6, 7]. One of the potential applications is automatic adverse drug reaction (ADR) identification from EMRs. The ADR terminology is an unpleasant event (e.g., symptom, disease, and finding) associated with a medication given at recommended dosages [8]. Even though ADRs can be identified by premarketing clinical trials, only partial ADRs are reported. Postmarketing surveillance with a large amount of population is necessary for remaining ADR monitoring. To this end, there are two multidisciplinary tasks of ADR surveillance: ADR identification and ADR prediction. The former task targets on retrieval of unrecognized ADR that may exist in data but not explicitly described as knowledge, while the latter one aims to construct a model for predicting unknown ADR that have not been reported in anywhere.

In earlier research, the statistical co-occurrence method is broadly employed to quantify the relationship strength between a drug-event pair. While the method is simple, its result might present no explicit clinical relevance of a derived drug-event pair [9] due to disregard relational context that might express an exact impression in a clinical event such as a drug treats a symptom or a drug causes a symptom. To fill in this research gap, many researchers consider surrounding contexts around drug and event entities within clinical texts and represent such data by either using pattern-based method [10–15] or feature-based method [16–18]. Consequently, a potential ADR is identified by either training supervised learning or semisupervised learning [19] model. However, there are two main difficulties when dealing with unstructured texts using such learning models. A rare availability of labeled instances derived by human annotation to form a gold-standard example is the former problem, and intractable processing of unstructured clinical texts is the latter one. Toward the insufficiency of labeled instances, several studies alleviate this problem by using a sort of heuristics or rules (distant supervision [20, 21]), that is, mapping a sentence that contains entity pair (*e*_1_, *e*_2_) from knowledge base and tagging relation label (*y*) to such mentioned sentence to form a training set. For the second problem, a word-based approach [22–24], the most commonly used method for text representation, is introduced; however, the method ignores either grammatical or semantic dependency among words. Therefore, pattern-based methods [10, 11, 14] are promoted to either extensive or substitute for word-based text representation. Recently, distant supervision paradigm is introduced to overcome hand-labeled data process to obtain a label of an instance from knowledge base [20, 21]. For example, knowledge bases consist of the following drug-event relations (“*ramipril-allergy*,” “ADR”) and (“*aspirin-fever*,” “IND”), so-called *entity-level* relation. By distant supervision, we can derive automatic labeled data of an associated sentence with such drug event, for example, “His *ramipril* were discontinued due to *allergy* and added to list in our medical records,” “ADR,” and known as *instance-level* relation. Therefore, multiple-instant learning (MIL) paradigm [25] is introduced into the classifier builder process to handle such two-level relations.

This paper introduces ADR identification framework by aiming to classify an *entity-level* relation of a drug-event pair. Our work differs from prior related works in the following aspects: (i) we propose key phrasal pattern-based bootstrapping method for characterizing ADR and IND, (ii) we introduce alternative parameter learning of a generative model, and (iii) we perform enhancement of the proposed method by incorporating transductive learning method.

The rest of this paper is organized as follows. A brief literature review and fundamental knowledge are given in Section 2. Then, Section 3 introduces problem formulation and our proposed framework.  Section 4 presents the experimental results. Finally, the conclusion is discussed in Section 5.

## 2. Background

### 2.1. Adverse Drug Reaction Identification from Unstructured Texts

Recently, narrative notes in EMRs have been demonstrated as a promising data source and widely utilized for improving detection of patients experiencing adverse reactions, across drugs and indication areas [10–13, 26]. There are at least three common subprocesses for dealing with unstructured texts in EMRs: (i) named entity recognition (NER) (particularly, named entities of drug and event) and normalization, (ii) relation generation (drug-event candidates), and (iii) relation classification (ADR identification).

**Figure 1:**
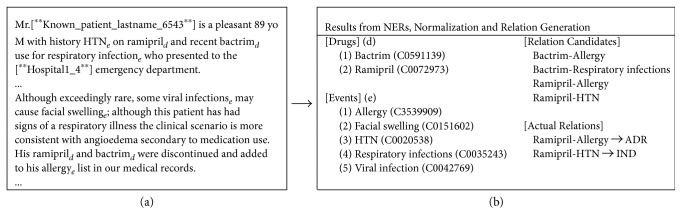
An example of narrative notes from a discharge summary in an EMR system is shown in (a). The possible outcomes derived by NERs, normalization, and relation generation of drugs and events from the given texts are displayed in (b). Both drugs and events are unified by UMLS CUI. For privacy concerns, confidential information is concealed using deidentification as [^∗∗^ … ^∗∗^].

As the first subprocess, the medical NER aims to recognize a clinical term mentioned in EMRs. Another extended task, the normalization intends to unify a discovered clinical term into a conventional lexicon based on an identical semantic meaning or a *concept*, which can be referred through *UMLS concept unique identifier (CUI)* (https://www.nlm.nih.gov). Many researchers endeavor to deal with medical NER and normalization by developing computational tools such as cTAKES (http://ctakes.apache.org), FreeLing-Med, MetaMap (https://metamap.nlm.nih.gov), MedLEE (http://www.medlingmap.org/taxonomy/term/80), tmChem (https://www.ncbi.nlm.nih.gov/CBBresearch/Lu/Demo/tmTools/tmChem.html), DNorm (https://www.ncbi.nlm.nih.gov/CBBresearch/Lu/Demo/tmTools/DNorm.html), GATE (https://gate.ac.uk), or Stanford CoreNLP tool (http://stanfordnlp.github.io/CoreNLP). From Figure 1, by employing medical NER and normalization, we can identify two drugs (i.e., *ramipril* and *bacterium*) and five events (i.e., *allergy, facial swelling, HTN (hypertension), respiratory infection,* and *viral infection*) from the given clinical texts. Then, the normalization task replaces a drug term or an event term with CUI. For example, a drug term *ramipril* is replaced with *C0072973*, or an event term *HTN* is replaced with *C0020538*, which refers to a concept of hypertension disease (NCI—https://nciterms.nci.nih.gov).

As the next subprocess, the generating of drug-event candidates is performed using the windowing technique [27–29]. A drug-event pair tends to form a relation if they are located in the same sentence, the same section, or more practically in the same window size *n*. In general, this boundary detection (BD) task aims to detect the beginning and the ending points within given texts that a drug and an event tend to be semantically related. The challenges of BD task [30–32] have arisen based on a boundary of interest and a domain of given texts. Many previous works define a potential boundary of a drug-event candidate within the same sentence, and the sentence boundary detection (SBD) in clinical texts is recognized as challenge with noise prone. One of the major issues is usage ambiguity of a *period* or a *full stop* (“.”). Typically, the *period* has several possible functions, such as a sentence boundary marker, a floating–point marker (e.g., “0.08,” “40.5 mg”), a marker for a numeric bullet of an enumerated list, or a separator within an abbreviation (e.g., “y.o.,” “h.s.”). Other punctuation marks such as a *colon* (“:”) increase the complexity of SBD as well. Additionally, the grammatical dependency is a potential method for improving a window-based relation generation because it considers more specific semantic dependency of the surrounding contexts.

Lastly, the generated lists of drug-event candidates are identified as ADR or IND using supervised, semisupervised, or unsupervised learning methods. The potential works on ADR identification from unstructured texts are summarized in Table 1. A statistical association is one of the pioneer works to identify ADR by considering the co-occurrence of a drug and an event in a specified window size *n* to form association hypotheses, and then, the 2 × 2 contingency table is computed for hypothesis testing. Despite the method is simple, it disregards semantic dependency among surrounding contexts that might express real clinical evident. On the other hand, a pattern-based method [14, 15] is manifested that achieves more accurate clinical relation extraction because it relies on cues or trigger words that usually implies a semantic relation. Although, a pattern-based method is more efficient than the window-based method, a set of predefined patterns or redundant pattern filtering by a human is required. In our previous work [13], a pattern-based method has been proposed to utilize labels weakly suggested by a set of simple rules, (distant supervision) and pattern distribution has been investigated for characterizing ADR relations. Different from [10–12, 18, 37], a pattern-based method is acquired as feature representation and machine learning methods such as support vector machine (SVM), decision tree C4.5 (DT), random forest (RF), or naïve Bayes (NB) are well-established as a classifier. Kang et al. [36] deploy a graph base and applies the shortest-path preference to ADR identification. With regard to the efficacy of word embedding [40] in NLP, Henriksson et al. [26] examine the distributional semantic model derived by word-embedding method for NER, concept attribute labeling, and relation classification. In their work, a high dimension on semantic space of each word is used as a feature for model learning. The distributional semantic model is shown to improve the classifier performance for all tasks. In another work, Nikfarjam et al. [17] apply the word embedding in a similar manner. However, to generalize semantic space, the authors employ a clustering method on such semantic vectors.

### 2.2. Distant Supervision and Multiple Instance Learning

The main objective of distant supervision is to alleviate the problem of hand-labeled training which is time-consuming, rare, and expensive/costly by relying on knowledge base. Such knowledge base is reliable, cheap, and ubiquitously available. Distant supervision is first introduced by Craven and Kumlien [20]. In their work, the term *weakly labeled data* is presented for biomedical relation extraction from MEDLINE. Lately, Mintz et al. [21] propose an interchangeable paradigm, *distant supervision*, to extract relation from Freebase. Their assumption relies on “if the two entities participate in a relation, any sentence that contains those two entities might express that relation.” The distant supervision has been applied recently for relation extraction problem [41–45] by mapping relations of any couple entities from knowledge bases (e.g., Freebase, YAGO) to a sentence in a large-scale text corpus (e.g., New York Times). Similarly, in previous works on application for emotion classification from social media (i.e., tweets, microblog text) [46–48], the authors make use of distant supervision to map lexicon emoticons or smilies from knowledge bases (i.e., Wikipedia, Weibo) to large-scale noisy texts. In medical domain, distant supervision for ADR identification [33, 49] is leveraged to automatically assign adverse reaction relation by mapping drug-event pair from knowledge bases to each health-related texts. The work of Yates et al. [49] utilizes SIDER as knowledge based on English tweets and posted messages from breast cancer forum, and Segura-Bedmar et al. [33] deploy SpanishDrugEffectDB database on Spanish health-related texts.

As mentioned in the previous section, applying distant supervision on text corpus mostly encounters the two-level relation concept and the entity-level and the instance-level relations. This mapping procedure may trigger noisy labeled data [50, 51], and MIL paradigm [25] is widely used as a solution [41, 42, 52, 53] for such wrongly labeled data problem. Fundamentally, MIL is aimed at handling the situation that training labels are associated with sets of instance examples rather than individual examples [54]. The concept of MIL considers two levels of data, namely *bag-* and *instance-level* relations. Let *𝒳* be an instance space, *𝒴* be a set of labels, where *𝒴* = {−1, +1}, and {(**x**_1_, *y*_1_), (**x**_2_, *y*_2_),…, (**x**_*n*_, *y*_*n*_)} be a training set, where **x**_*i*_ ∈ *𝒳* is an instance and *y*_*i*_ ∈ *𝒴* is a known label of **x**_*i*_; usually, the supervised learning is to train a classifier function *f* : *𝒳* → *𝒴*. On the one hand, a given training set in MIL consists of bags and bag labels as {(*B*_1_, *y*_1_), (*B*_2_, *y*_2_),…, (*B*_*n*_, *y*_*n*_)}, where *B*_*i*_ = {**x**_*i*1_, **x**_*i*2_,…, **x**_*im*_} is a set of multiple instances, **x**_*ij*_ ∈ *𝒳*, and *y*_*i*_ ∈ *𝒴* is a label of bag *B*_*i*_ and *m* can be different across a particular bag, the goal of MIL is to learn *f* : 2^*𝒳*^ → *𝒴*. For ADR identification problem, bag- and instance-level relations in MIL are equivalent to the entity- and the instance-level relations of drug-event relation by distant supervision, respectively.

### 2.3. Transductive Learning

In semisupervised learning, as varieties of the prediction method, the three parameters are (i) predictive model, (ii) single model or collaborative model, and (iii) test instances handling model. As the first parameter, recent works [55–57] have proposed various predictive models, such as generative models [22, 58], low-density separation models [59], and graph-based models [60]. For the second parameter, at least two alternatives, namely self-training [61, 62] or cotraining [63], can be applied to assign a label to an unlabeled instance by either one single predictive model or multiple ensemble predictive models. The last parameter concerns with how to handle test instances, where two choices are (i) to manipulate the test instances separately from the unlabeled instances (inductive learning) or (ii) to treat them as unlabeled instances in the training step (transductive learning). Regardless of any choice for the above three parameters, semisupervised learning requires a few labeled instances for constructing an initial model, triggering complexity in the acquisition of such initial labeled data. The main idea of transductive learning is to take advantage of the information from unlabeled data during training time, while inductive learning ignores such information even though they are available [19].

## 3. Methods

**Figure 2:**
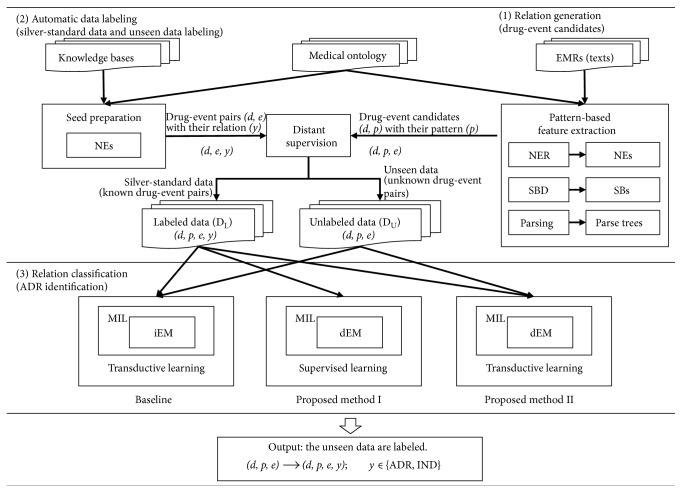
Our ADR identification framework consists of the three main tasks. (1) In the relation generation, drug-event pairs (*d*, *e*) are extracted from a corpus together with their patterns (*p*) using named entity recognition (NER), sentence boundary detection (SBD), and parsing. (2) In the automatic data labeling, distant supervision assigns a relation label (*y*) to each drug-event pair (*d*, *e*) obtained from the relation generation with its pattern (*p*) if such relation exists in knowledge base. The *silver-standard* data set is labeled data in the experiment. Here, two types of output data sets are a set of labeled data (*𝒟*_L_), composed of (*d*, *p*, *e*, *y*) extracted from a corpus (EMR texts), where the labels (*y*) are defined for the drug-event pairs (*d*, *e*) in the knowledge base, and a set of unlabeled data (*𝒟*_U_), composed of (*d*, *p*, *e*) extracted from a corpus, where the labels do not exist for the drug-event pairs (*d*, *e*) in the knowledge base. (3) In this relation classification, this work proposes three types of generative models with independent/dependent expectation-maximization (EM) model (iEM/dEM): (i) transductive learning with iEM (baseline), (ii) supervised learning with dEM, and (iii) transductive learning with dEM.

This section presents the proposed ADR identification framework to overcome the shortcomings of the existing research: (i) the lack of domain experts for instances labeling and (ii) intractable processing of large-scale unstructured clinical texts. Our proposed framework contains the three main tasks (Figure 2). First, a set of drug-event candidates is generated from EMR texts. A silver-standard data and unseen data preparation are the next process. Finally, we explore alternative parameter learning schemes of generative models to identify potential drug-event relations.

To solve the first issue, we assign a label to an unlabeled instance by exploiting facts in knowledge bases (i.e., SIDER and DrugBank) and consider two labels, ADR and IND, as classification outputs. While distant supervision can supply a label to an unlabeled instance by simply looking up from knowledge bases, the labeled data set by this method is formed as MIL problem which training labels are associated with sets of instance examples rather than individual examples. As for the latter issue, applying phrase-based method and dependency representation may improve the model performance. In our work, the main idea is that a sentence regarding harmful (ADR) or beneficial (IND) clinical events can be simplified into the three key elements, *a drug*, *a key phrasal pattern*, and *an event*, and dependency among such three elements has significance. Such key phrasal pattern implies a semantic relation between any pair of drug and event entities. We have employed key phrasal pattern-based method for ADR identification in our previous work [13]. The method exhibits the high precision; notwithstanding its drawback is low recall rate due to a limit to the number of key phrasal patterns and the utilization of simple models. In this work, we extend such key phrasal pattern-based method with more sophisticated models, which is expected to be able to retain the high precision and improve retrieval performance. The EM, an iterative method, is incorporated with Markov property assumption to draw conditional probability distribution of pattern-based feature (dEM). Finally, we leverage unlabeled data through the transductive learning as semisupervised learning to enhance the performance of the proposed framework. For performance evaluation, we construct EM with independent assumption through NB (iEM) as the baseline and also compare our proposed methods to multiple advanced methods; multiple-instance support vector machine (MISVM), multiple-instance naïve Bayes (MINB), multiple-instance logistic regression (MILR), and transductive support vector machine (TSVM). The multiple numbers of parameters such as pattern types, pattern-weighting models, and initial and iterative weighting relation labels for unlabeled data are investigated throughout three alternative MIL models: iEM with transductive learning setting (baseline), dEM-supervised learning, and dEM with transductive learning.

### 3.1. Problem Formulation

We firstly present the formal definition of distant supervision and then formulate the problem using MIL concept. Let *𝒦* denote knowledge bases regarding ADR and IND that are obtained from SIDER (http://sideeffects.embl.de) and DrugBank (https://www.drugbank.ca), *𝒯* be a set of seeds, where *𝒯*⊆*𝒦*, and *𝒴* is a set of labels, where *𝒴* = {ADR, IND}; the data set of seeds *𝒯* in knowledge bases *𝒦* or an *entity-level* set can be defined as *𝒯* = {(**t**_1_, *y*_1_), (**t**_2_, *y*_2_),…, (**t**_*N*_, *y*_*N*_)}, where **t**_*i*_ = {*d*_*i*_, *e*_*i*_} is a seed, **t**_*i*_ ∈ *𝒢* is 2-dimensional entities space which consists of a drug entity (*d*_*i*_) and an event entity (*e*_*i*_) that are defined in *𝒦*, *y*_*i*_ ∈ *𝒴* is a label corresponding **t**_*i*_, and *N* is a total number of seeds. Therefore, the data set of seeds can be derived as *𝒯* = {(*d*_1_, *e*_1_, *y*_1_), (*d*_2_, *e*_2_, *y*_2_),…, (*d*_*n*_, *e*_*n*_, *y*_*n*_)}. For instance, the drug *ramipril* associates with the adverse event *allergy* and the drug *ibuprofen* is used to treat the event *arthritis* as a symptom which is supposed to exist in *𝒦*. We can derive a data set of seeds to be a source of distant supervision as *𝒯* = {(ramipril_*d*_, allergy_*e*_, ADR), (ibuprofen_*d*_, arthritis_*e*_, IND)}. These seeds are *entity-level* data that are used as knowledge for later processes.

Let *𝒞* be a clinical-record corpus from MIMIC (https://mimic.physionet.org), which contains a set of discharge summary sentences *𝒮*. We transform each sentence into the three key elements, that is, a drug entity (*d*), a key phrasal pattern entity (*p*), and an event entity (*e*), while semantic of such simplified texts is retrained. Given **x**_*j*_ = {*d*_*j*_, *p*_*j*_, *e*_*j*_} is a tuple obtained from an input sentence and **x**_*j*_ ∈ ℋ is 3-dimensional entity space, in order to automatically generate labeled examples using distant supervision, the goal is to obtain a mapping function *f* : ℋ → *𝒴* that relates a drug-event pair of {*d*_*j*_, *e*_*j*_} to a relation label *y*_*i*_, where (*d*_*i*_, *e*_*i*_, *y*_*i*_) exists in *𝒯*, *d*_*j*_ = *d*_*i*_, and *e*_*j*_ = *e*_*i*_. Finally, we can derive a set of labeled data *𝒟*_L_ = {(*d*_1_, *p*_1_, *e*_1_, *y*_1_), (*d*_2_, *p*_2_, *e*_2_, *y*_2_),…, (*d*_*n*_, *p*_*n*_, *e*_*n*_, *y*_*n*_)}, namely, an *instance-level* data set, whereas *n* is a total number of mapped sentences.

For example, the sentence “His ramipril were discontinued due to allergy and added to list in our medical records.” is supposed to exist in the corpus *𝒞*. Then, the transformed sentence **x**_1_ using a dependency tree can be simplified into the three key elements of a drug *d*_1_ = {ramipril_*d*_}, a key phrasal pattern *p*_1_ = {be‐discontinue‐due‐to_*p*_}, and an event *e*_1_ = {allergy_*e*_}, where a key phrasal pattern is applied in either the syntactically lemmatized lexicon or surface lexicon (e.g., was-discontinued-due-to), and can be employed as either word or phrase form (discuss later in Section 3.3.1). From the mapping function *f* : ℋ → *𝒴*, we can project such sentence **x**_1_ to a seed {(ramipril_*d*_, allergy_*e*_, ADR)} in *𝒯* and transfer corresponding labels ADR to the sentence **x**_1_. Therefore, we can derive a labeled data by distant supervision as {(ramipril_*d*_, be‐discontinue‐due‐to_*p*_, allergy_*e*_, ADR)} ∈ *𝒟*_L_. As another example, a sentence “The allergy improved despite ongoing treatment with ramipril.” is also supposed to exist in the corpus *𝒞*. The transformed sentence **x**_2_ is {ramipril_*d*_, improved‐despite_*p*_, allergy_*e*_}. In the similar manner, we can use the mapping function *f* : ℋ → *𝒴* to assign the corresponding label of the entity pair ramipril_*d*_ and allergy_*e*_. Therefore, the derived labeled data is {ramipril_*d*_, improved‐despite_*p*_, allergy_*e*_, ADR} ∈ *𝒟*_ℒ_. However, the sentence **x**_2_ might not express the correctly clinical event of adverse reaction. This is known as the noisy label and need to to be handled by a particular technique such as MIL.

In MIL concept, bag- and instance-level relations are equivalent to the entity- and the instance-level relations of drug-event relation derived by distant supervision, respectively. Regarding the definition in Section 2.2, *𝒳* is an instance space, *𝒴* is a set of labels, where *𝒴* = {ADR, IND}, the labeled data set *𝒟*_L_ can be rewritten in the form of MIL as *𝒟*_L_ = {(*B*_1_, *y*_1_), (*B*_2_, *y*_2_),…, (*B*_*n*_, *y*_*n*_)}, where *B*_*i*_ = {**x**_*i*1_, **x**_*i*2_,…, **x**_*im*_} is a set of multiple sentences which all sentences in a bag *B*_*i*_ correspond to the same drug (*d*) and event (*e*), *n* is the number of bags, and *m* is the number of sentences in a bag and can be varied across a different bag. On the one hand, unlabeled instances (*𝒟*_U_) are formed as a group of bags in the similar way but without a label as *𝒟*_U_ = {(*B*_1_), (*B*_2_),…, (*B*_*n*_)}. Our goal is both to train an instance classifier function *f* : *𝒳* → *𝒴* in the instance–space paradigm from *𝒟*_L_ only (supervised learning) and attempt to infer the accurate label for each instance in the *𝒟*_U_ set during the training process (transductive learning). The bag label, eventually, can be derived from an aggregation function of the instance level, and the model assessment is investigated through the model performance of the entity level. Regarding noisy data labeling from distant supervision, the collective assumption and standard assumption with logical-OR aggregation for the bag label judgment are rather improper. The relaxed version of the MIL standard assumption is used in our proposed framework by assuming that the positive and negative bags are able to contain a mixture of either positive or negative instances, but the probability of *at least one* positive instance should be the maximum for the positive bag and vice versa. Consequently, to learn bag classifier *f* : 2^*𝒳*^ → *𝒴*, the estimated bag label from an instance classifier can be computed using (1), where *y*_*i*_ is a label of a bag *i* (the entity-level label), *y*_*ij*_ is a label of the instance-level and possibly different for each sentence instance *j* within the same bag *i*, and *n* is the total number of sentences in the bag.

(1) $$\begin{array}{c} p\left(y_{i}\;|\;B_{i}\right)=\underset{j\in \left\{1,\ldots ,n\right\}}{\max}p\left(y_{ij}\;|\;x_{ij}\right). \end{array}$$

Generally, the training data are not sufficient for parameter training. In order to learn such classifier function *f* : *𝒳* → *𝒴*, we make use of the iterative EM technique with transductive learning setting to estimate the posterior probability *p*(*y* | **x**) through the two parameters, that is, prior probability *p*(*y*) and class-conditional density *p*(**x** | *y*), of the generative model.

### 3.2. Medical Named Entity Recognition and Relation Candidate Generation

**Figure 3:**
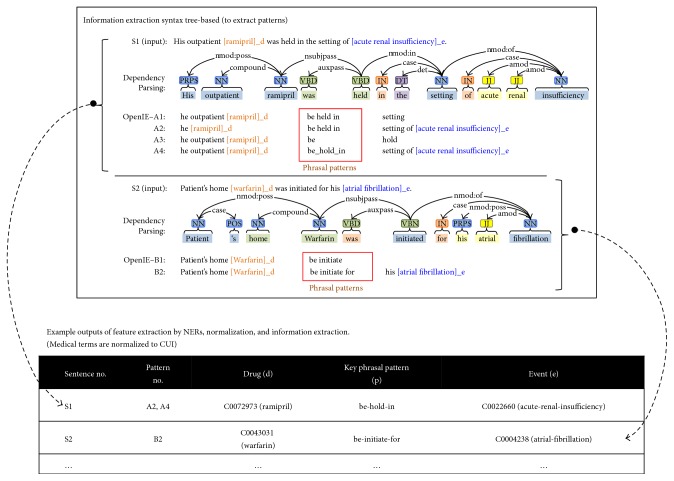
The upper block depicts the dependency parsing of two sentences (S1 and S2) and their outputs from OpenIE. The lower table exhibits their final representations in the form of a relational table. Generally speaking, this syntactic-based analyzer extracts a list of drug-key phrasal pattern-event tuples from the sentences, where drugs and events are matched with their corresponding CUIs.

Figure 3 displays information extraction from sentences in the MIMIC corpus, with the output of drug-key phrasal pattern-event tuples as candidates of ADR or IND relation. This process involves NER, SBD, and parsing. Here, the MetaMap [64] is used for NER, our in-house program for SBD (https://github.com/makoto404/MIMIC_SBD), and Stanford CoreNLP's OpenIE for parsing. After extracting relation candidate tuples (entity_1_, predicate, entity_2_), we select only the tuples that include drug name and event name as entity_1_ and entity_2_ or vice versa. The output is in the form of (a drug, a key phrasal pattern, and an event).

**Figure 4:**
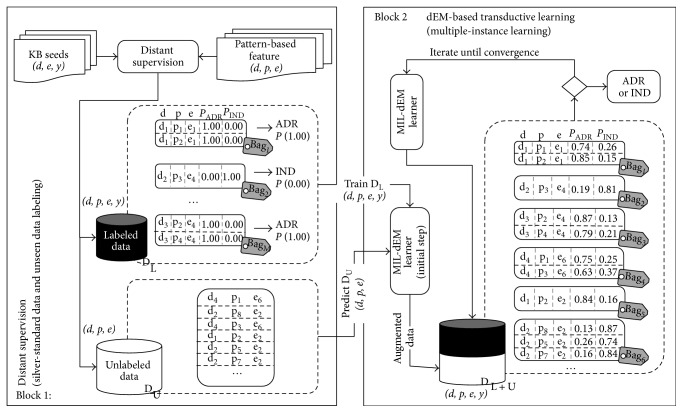
Block 1 expresses the data labeling using the fact from external sources (KB seeds). The *𝒟*_L_ is a data set that a pair of drug and event entities can be mapped to a set of KB seeds through the distant supervision. Hence, all sentences that correspond to the same drug-event pair are assigned to the same bag and same label (labeled data *𝒟*_L_) regarding a label of such drug-event pair in a set of seeds from knowledge base. Finally, such *𝒟*_L_ set is used as a training data. Block 2 depicts our proposed MIL-dEM method. The label assignment for unlabeled *𝒟*_U_ data set (test set) can be obtained from a classifier in the previous process. Lastly, such unlabeled data is incorporated and contributed to estimating the parameters of a generative model.

The automatic labeling process using distant supervision is illustrated in Figure 4. Firstly, each pair of drug and event (*d*, *e*) from the set of seeds in knowledge bases is used to extract drug-event pairs from the set of sentences; then, we assign the label corresponding to the seed label to all sentences that mention such (*d*, *e*) pair. However, to reduce the ambiguity of the ground truth from knowledge base supervision, a pair of (*d*, *e*) that is found to exhibit both of ADR and IND semantic relations is excluded. Given a set of sentences *𝒳*, the training set *𝒟*_L_ is in the form {(*B*_1_, *y*_1_), (*B*_2_, *y*_2_),…, (*B*_*n*_, *y*_*n*_)}, where *B*_*i*_ = {**x**_*i*1_, **x**_*i*2_,…, **x**_*im*_}. In the Block 1 of Figure 4, the first bag (Bag_1_) consists of two sentences that correspond to the same entity-level of drug *d*_1_ and event *e*_1_. The second bag (Bag_2_) contains only one sentence relevant to drug *d*_2_ and event *e*_4_.

Finally, all sentences that are able to be assigned a label by distant supervision are referred as the set of labeled data *𝒟*_L_ and the remaining data that are not matched by distant supervision is used as unlabeled data *𝒟*_U_.

### 3.3. Document Representation

#### 3.3.1. Feature Extraction for Clinical Textual Data

To recognize a relation between a drug and an event, our approach generates a set of relation candidates (drug-event pairs) from medical records in the form of (drug, pattern, event).  Table 2 depicts examples of multiple types of feature extraction and drug-event candidates. Our work considers two parameters related to representing such relation candidates. The first parameter, called relation boundary constraint, defines potential of using surrounding context for determining drug-event relations while the second and third parameters, called syntactic lemmatization and pattern granularity constraints, are related to patterns used to detect drug-event relations, as follows.

- *Syntactic lemmatization*: for syntactic word forms, two possibilities are syntactically lemmatized lexicons (*L*) and surface lexicons (*S*).
- *Pattern granularity*: in terms of pattern units, two options are in word form (*W*) and phrase form (*P*).

#### 3.3.2. Pattern-Weighting Models

(i) *Bernoulli (binary) document model*(*B*): a document (hereinafter referred to as a sentence denoted by *x*) can be represented in the form of a vector each element of which corresponds to a term (i.e., word, phrase) denoted by *w* with a value of either 0 or 1 for presence or absence of such term, respectively.
  (2) $$\begin{array}{c} x_{B}=\left\{B\left(x,w_{1}\right),B\left(x,w_{2}\right),\ldots ,B\left(x,w_{\left|W\right|}\right)\right\}, \end{array}$$
  where **x**_*B*_ presents a sentence *x* in the form of a binary vector, *B*(*x*, *w*_*i*_) = 1 when *w*_*i*_ is the *i*th term in the sentence *x* (otherwise 0), and *w*_*i*_ is a term in the universe *W*.
(ii) *Multinomial (frequency) document model*: a sentence is expressed by a vector of term frequency (TF) as
  (3) $$\begin{array}{c} x_{TF}=\left\{TF\left(x,w_{1}\right),TF\left(x,w_{2}\right),\ldots ,TF\left(x,w_{\left|W\right|}\right)\right\};\\ TF\left(x,w_{i}\right)=\frac{f_{x}\left(w_{i}\right)}{\left|x\right|}, \end{array}$$
  where **x**_TF_ is a sentence *x* in the form of a TF vector, TF(*x*, *w*_*i*_) expresses the normalized frequency of the *i*th term *w*_*i*_ by the sentence size |*x*|, and *f*_*x*_(*w*_*i*_) is the frequency that the term *w*_*i*_ occurs in the sentence *x*. As another option, a document can also be expressed by a vector of term frequency-inverse document frequency TFIDF as
  (4) $$\begin{array}{c} x_{TFIDF}=\left\{TF\left(x,w_{1}\right)\cdot IDF\left(w_{1}\right),TF\left(x,w_{2}\right)\cdot IDF\left(w_{2}\right),\ldots ,TF\left(x,w_{\left|W\right|}\right)\cdot IDF\left(w_{\left|W\right|}\right)\right\};\\ TF\left(x,w_{i}\right)=\frac{f_{x}\left(w_{i}\right)}{\;|\;x\;|\;};IDF\left(w_{i}\right)=\log\frac{\left|X\right|}{\left|\left\{x\;|\;x\in X,\;B\left(x,w_{i}\right)=1\right\}\right|}, \end{array}$$
  where **x**_TFIDF_ is a sentence *x* ∈ *𝒳* (the document universe), in the form of a TFIDF vector, and IDF(*w*_*i*_) expresses the inverse document frequency, corresponding to the logarithm of the ratio of the total number of sentences in the universe |*𝒳*| to the number of sentences that contain the *i*th term *w*_*i*_.

### 3.4. Probabilistic Classification Modeling

This section describes two EM-based probabilistic classification models, one with independent assumption (iEM) and the other with dependent representation assumption (dEM).

#### 3.4.1. EM Model with Naïve Bayes Independent Assumption (iEM)

Let *𝒳* = {**x**_1_, **x**_2_,…, **x**_|*𝒳*|_} be a set of sentences, **x**_*i*_ = {*w*_*i*1_, *w*_*i*2_,…, *w*_*i*|*𝒳*_*i*_|_} be a sentence that includes |**x**_*i*_| terms, and *C* = {*c*_1_, *c*_2_,…, *c*_|*C*|_} be the set of possible classes. The probability that the sentence **x**_*i*_ has *c*_*k*_ as its class (*y*_*i*_ = *c*_*k*_) can be formulated as

(5) $$\begin{array}{c} p\left(y_{i}=c_{k}\;|\;x_{i}\right)=\frac{p\left(c_{k}\right)\;p\left(x_{i}\;|\;c_{k}\right)}{p\left(x_{i}\right)}=\frac{p\left(c_{k}\right)\;p\left(x_{i}\;|\;c_{k}\right)}{\sum_{k=1}^{\left|C\right|}p\left(c_{k}\right)p\left(x_{i}\;|\;c_{k}\right)}. \end{array}$$

While in most situations, it is possible to obtain the class *p*(*c*_*k*_) simply from the training set, and the generative probability of **x**_*i*_ given a class *c*_*k*_ usually suffers with insufficient training data. As done by several works, the assumption of independence, usually called naïve Bayes (NB), can be applied to alleviate this sparseness problem as expressed in

(6) $$\begin{array}{c} p\left(x_{i}\;|\;c_{k}\right)=p\left(w_{i1},w_{i2},\ldots ,w_{i\left|x_{i}\right|}\;|\;c_{k}\right)=p\left(w_{i1}\;|\;c_{k}\right)\cdot p\left(w_{i2}\;|\;w_{i1},c_{k}\right)\cdot \;\ldots \;\cdot p\left(w_{i\;|\;x_{i}\;|\;}\;|\;w_{i1},w_{i2},\ldots ,w_{i\left(\left|x_{i}\right|-1\right)},c_{k}\right)\approx p\left(w_{i1}\;|\;c_{k}\right)\cdot p\left(w_{i2}\;|\;c_{k}\right)\cdot \;\ldots \;\cdot p\left(w_{i\left|x_{i}\right|}\;|\;c_{k}\right)=\overset{\left|x_{i}\right|}{\underset{j=1}{\prod }}p\left(w_{ij}\;|\;c_{k}\right). \end{array}$$

Therefore, the NB text classifier can be rewritten in the form

(7) $$\begin{array}{c} p\left(y_{i}=c_{k}\;|\;x_{i}\right)=\frac{p\left(c_{k}\right)\;\prod_{j=1}^{\left|x_{i}\right|}p\left(w_{ij}\;|\;c_{k}\right)}{\sum_{k=1}^{\left|C\right|}p\left(c_{k}\right)\;\prod_{j=1}^{\left|x_{i}\right|}p\left(w_{ij}\;|\;c_{k}\right)}. \end{array}$$

Here, it is necessary to estimate two sets of parameters, denoted by *θ*, of expectation-maximization (EM) algorithm. The first parameter set is the class-conditional probability of any term *w*_*q*_ ∈ *W* given the class *c*_*k*_ while the other one is the probability of the class *c*_*k*_. The parameter set is defined by

(8) $$\begin{array}{c} \theta =\left\{p^{\left(t+1\right)}\left(w_{q}\;|\;c_{k}\right),p^{\left(t+1\right)}\left(c_{k}\right)\right\}. \end{array}$$

In the expectation step (E-step), for each iteration, the *θ* parameter of the previous step is applied to re-estimate the model probability. In our experiment, the convergence threshold is 10^−7^ and the maximum number of iterations is set to 50.

(9) $$\begin{array}{c} p^{\left(t\right)}\left(y_{i}=c_{k}\;|\;x_{i}\right)=\frac{p^{\left(t-1\right)}\left(c_{k}\right)\;\prod_{j=1}^{\left|x_{i}\right|}p^{\left(t-1\right)}\left(w_{ij}\;|\;c_{k}\right)}{\sum_{k=1}^{\left|C\right|}p^{\left(t-1\right)}\left(c_{k}\right)\;\prod_{j=1}^{\left|x_{i}\right|}p^{\left(t-1\right)}\left(w_{ij}\;|\;c_{k}\right)} \end{array}$$

For the maximization step (M-step), with a Laplace smoothing factor *λ* > 0, the (*t* + 1)th-iteration probability of *p*^(*t* + 1)^(*w*_*q*_ | *c*_*k*_) and *p*^(*t* + 1)^(*c*_*k*_) can be estimated from the *t*th-iteration probability. The maximum likelihood estimation for NB is simply computed from an empirical corpus using

(10) $$\begin{array}{c} p^{\left(t+1\right)}\left(w_{q}\;|\;c_{k}\right)=\frac{\lambda +\sum_{i=1}^{\left|X\right|}N\left(w_{q},x_{i}\right)p^{\left(t\right)}\left(y_{i}=c_{k}\;|\;x_{i}\right)}{\lambda \left|W\right|+\sum_{r=1}^{\left|W\right|}\sum_{i=1}^{\left|X\right|}N\left(w_{z},x_{i}\right)p^{\left(t\right)}\left(y_{i}=c_{k}\;|\;x_{i}\right)}, \end{array}$$

where *W* is a total number of terms and any term *w*_*z*_ ∈ *W*.

(11) $$\begin{array}{c} p^{\left(t+1\right)}\left(c_{k}\right)=\frac{\lambda +\sum_{i=1}^{\left|X\right|}p^{\left(t\right)}\left(y_{i}=c_{k}\;|\;x_{i}\right)}{\lambda \left|C\right|+\left|X\right|}. \end{array}$$

The following demonstrates an example of applying the above formulations with the key phrasal pattern-based feature. Given the *L–P* feature representation of **x**_*i*_ = (C0033487, be-hold-due-to, C0020649) corresponds to relation tuple (*d*_*i*_, *p*_*i*_, *e*_*i*_) obtained from an input sentence where the pattern *p*_*i*_ be the phrase form, we can estimate *p*(*y*_*i*_ = *c*_*k*_ | **x**_*i*_) as expressed in

(12) $$\begin{array}{c} p^{\left(t\right)}\left(y_{i}=c_{k}\;|\;x_{i}\right)=\frac{p^{\left(t-1\right)}\left(c_{k}\right)\cdot p^{\left(t-1\right)}\left(C0033487\;|\;c_{k}\right)\cdot p^{\left(t-1\right)}\left(be‐hold‐due‐to\;|\;c_{k}\right)\cdot p^{\left(t-1\right)}\left(C0020649\;|\;c_{k}\right)}{\sum_{k=1}^{\left|C\right|}p^{\left(t-1\right)}\left(c_{k}\right)\cdot p^{\left(t-1\right)}\left(C0033487\;|\;c_{k}\right)\cdot p^{\left(t-1\right)}\left(be‐hold‐due‐to\;|\;c_{k}\right)\cdot p^{\left(t-1\right)}\left(C0020649\;|\;c_{k}\right)}. \end{array}$$

Another example, given the *L–W* feature representation of the same sentence **x**_*i*_ = {C0033487, be, hold, due, to, C0020649}, corresponds to relation tuple (*d*_*i*_, *p*_*i*_, *e*_*i*_) where the pattern *p*_*i*_ is in the word form. We can compute the class probability of the given texts *p*(*y*_*i*_ = *c*_*k*_ | **x**_*i*_) as

(13) $$\begin{array}{c} p^{\left(t\right)}\left(y_{i}=c_{k}\;|\;x_{i}\right)=\frac{p^{\left(t-1\right)}\left(c_{k}\right)\cdot p^{\left(t-1\right)}\left(C0033487\;|\;c_{k}\right)\cdot p^{\left(t-1\right)}\left(be\;|\;c_{k}\right)\cdot p^{\left(t-1\right)}\left(hold\;|\;c_{k}\right)\cdot p^{\left(t-1\right)}\left(due\;|\;c_{k}\right)\cdot p^{\left(t-1\right)}\left(to\;|\;c_{k}\right)\cdot p^{\left(t-1\right)}\left(C0020649\;|\;c_{k}\right)}{\sum_{k=1}^{\left|C\right|}p^{\left(t-1\right)}\left(c_{k}\right)\cdot p^{\left(t-1\right)}\left(C0033487\;|\;c_{k}\right)\cdot p^{\left(t-1\right)}\left(be\;|\;c_{k}\right)\cdot p^{\left(t-1\right)}\left(hold\;|\;c_{k}\right)\cdot p^{\left(t-1\right)}\left(due\;|\;c_{k}\right)\cdot p^{\left(t-1\right)}\left(to\;|\;c_{k}\right)\cdot p^{\left(t-1\right)}\left(C0020649\;|\;c_{k}\right)}. \end{array}$$

#### 3.4.2. EM Model with Dependency Representation (dEM)

We introduce a dependency representation as an alternative model representation that is based on the same intuitions as the NB model but less restriction regarding the implicitly strong independence assumptions. This dependency representation is an efficient factorization of the join probability distributions over a set of three random variables *w*_*q*_, *w*_*r*_, and *w*_*s*_, where each variable is a domain of possible values, that is, drug, key phrasal pattern, and event. We extend the dependency representation with iterative learning by EM approach in order to align the model assumption to the natural language and also figure out an unseen random variable using probability estimation based on an existing prior knowledge. This dependency representation is also known as Bayesian networks (BN) and the conditional probability of independent variable given a class probability can be derived by the chain rule

(14) $$\begin{array}{c} p\left(x_{i}\;|\;c_{k}\right)=p\left(w_{iq},w_{ir},w_{is}\;|\;c_{k}\right)=p\left(w_{iq}\;|\;c_{k}\right)\cdot p\left(w_{ir}\;|\;w_{iq},c_{k}\right)\cdot p\left(w_{is}\;|\;w_{iq},w_{ir},c_{k}\right). \end{array}$$

Therefore, the BN text classifier can be rewritten in the form

(15) $$\begin{array}{c} p\left(y_{i}=c_{k}\;|\;x_{i}\right)=\frac{p\left(c_{k}\right)\cdot p\left(w_{iq}\;|\;c_{k}\right)\cdot p\left(w_{ir}\;|\;w_{iq},c_{k}\right)\cdot p\left(w_{is}\;|\;w_{iq},w_{ir},c_{k}\right)}{\sum_{k=1}^{\left|C\right|}p\left(c_{k}\right)\cdot p\left(w_{iq}\;|\;c_{k}\right)\cdot p\left(w_{ir}\;|\;w_{iq},c_{k}\right)\cdot p\left(w_{is}\;|\;w_{iq},w_{ir},c_{k}\right)}. \end{array}$$

According to the core of BN representation, a random variable is represented by a node in a directed acyclic graph (DAG), and an edge between any two nodes is presented by an arrow line which implies a direct influence of one node on another node. Given a sentence **x**_*i*_ with three elements (*w*_*iq*_, *w*_*ir*_, and *w*_*is*_) in the form of a relation tuple (*d*_*i*_, *p*_*i*_, *e*_*i*_), there are three factorized ways (3!) as alternative model skeletons of the dependency representation through the chain rule. We, hence, propose the linear interpolation in order to weigh and combine the probability estimation from all of possible dependency representations as defined by

(16) $$\begin{array}{c} p\left(x_{i}\;|\;c_{k}\right)=p\left(w_{iq},w_{ir},w_{is}\;|\;c_{k}\right)\approx \gamma_{1}\left[p\left(w_{iq}\;|\;c_{k}\right)\cdot p\left(w_{ir}\;|\;w_{iq},c_{k}\right)\cdot p\left(w_{is}\;|\;w_{iq},w_{ir},c_{k}\right)\right]+\gamma_{2}\left[p\left(w_{iq}\;|\;c_{k}\right)\cdot p\left(w_{is}\;|\;w_{iq},c_{k}\right)\cdot p\left(w_{ir}\;|\;w_{iq},w_{is},c_{k}\right)\right]+\gamma_{3}\left[p\left(w_{ir}\;|\;c_{k}\right)\cdot p\left(w_{iq}\;|\;w_{ir},c_{k}\right)\cdot p\left(w_{is}\;|\;w_{iq},w_{ir},c_{k}\right)\right]+\gamma_{4}\left[p\left(w_{ir}\;|\;c_{k}\right)\cdot p\left(w_{is}\;|\;w_{ir},c_{k}\right)\cdot p\left(w_{iq}\;|\;w_{ir},w_{is},c_{k}\right)\right]+\gamma_{5}\left[p\left(w_{is}\;|\;c_{k}\right)\cdot p\left(w_{iq}\;|\;w_{is},c_{k}\right)\cdot p\left(w_{ir}\;|\;w_{iq},w_{is},c_{k}\right)\right]+\gamma_{6}\left[p\left(w_{is}\;|\;c_{k}\right)\cdot p\left(w_{ir}\;|\;w_{is},c_{k}\right)\cdot p\left(w_{iq}\;|\;w_{ir},w_{is},c_{k}\right)\right], \end{array}$$

such that the total *γ* is ∑_*i*=1_^6^*γ*_*i*_ = 1.

Generally, the linear interpolation method of three random variables can be estimated from the combination of two random variables and individual random variable. Similarly, two random variables are able to approximate from individual random variable as well. For instance, given two history terms *w*_*iq*_ and w*_ir_* in a sentence **x**_*i*_, the interpolation is comparatively estimated from individual random variable and two random variables as shown in

(17) $$\begin{array}{c} p\left(w_{ir}\;|\;w_{iq},c_{k}\right)=\beta_{1}p\left(w_{ir}\;|\;c_{k}\right)+\beta_{2}p\left(w_{ir}\;|\;w_{iq},c_{k}\right), \end{array}$$

such that the total *β* is ∑_*i*=1_^2^*β*_*i*_ = 1.

Another instance, three history terms (*w*_*iq*_, *w*_*ir*_, *w*_*is*_) in a sentence **x**_*i*_ are given; the likelihood estimation as shown in (18) can be derived similarly as the previous estimator by interpolation of individual random variable, two random variables, and three random variable estimators.

(18) $$\begin{array}{c} p\left(w_{is}\;|\;w_{iq},w_{ir},c_{k}\right)=\alpha_{1}p\left(w_{is}\;|\;c_{k}\right)+\alpha_{2}p\left(w_{is}\;|\;w_{iq},c_{k}\right)+\alpha_{3}p\left(w_{is}\;|\;w_{ir},c_{k}\right)+\alpha_{4}p\left(w_{is}\;|\;w_{iq},w_{ir},c_{k}\right), \end{array}$$

such that the total *α* is ∑_*i*=1_^4^*α*_*i*_ = 1.

Finally, we compute *p*(*w*_*iq*_ | *w*_*ir*_, *c*_*k*_), *p*(*w*_*iq*_ | *w*_*is*_, *c*_*k*_), *p*(*w*_*ir*_ | *w*_*is*_, *c*_*k*_), *p*(*w*_*is*_ | *w*_*iq*_, *c*_*k*_), and *p*(*w*_*is*_ | *w*_*ir*_, *c*_*k*_) with the similar manner as (17) and calculate *p*(*w*_*iq*_ | *w*_*ir*_, *w*_*is*_, *c*_*k*_) and *p*(*w*_*ir*_ | *w*_*iq*_, *w*_*is*_, *c*_*k*_) using the same way as shown in (18).

In the same manner as the NB model, it is necessary to estimate the four sets of parameters *θ* whereas any terms *w*_*q*_, *w*_*r*_, *w*_*s*_ ∈ *W*.

(19) $$\begin{array}{c} \theta =\left\{p^{\left(t+1\right)}\left(w_{q}\;|\;c_{k}\right),p^{\left(t+1\right)}\left(w_{q}\;|\;w_{r},c_{k}\right),p^{\left(t+1\right)}\left(w_{q}\;|\;w_{r}w_{s},c_{k}\right),p^{\left(t+1\right)}\left(c_{k}\right)\right\}. \end{array}$$

The iterative learning using EM approach is applied to estimate the parameter *θ*. For the E-step, for each iteration, the *θ* parameter is applied to re-estimate the model probability as shown in (20) and (21). This process will repeat until convergence. The same setting as the iEM model, the value of 10^−7^ for the convergence threshold and the value of 50 for the maximum number of iterations, is applied for dEM model as well.

(20) $$\begin{array}{c} p^{\left(t-1\right)}\left(x_{i}\;|\;c_{k}\right)\approx \gamma_{1}\left[p^{\left(t-1\right)}\left(w_{iq}\;|\;c_{k}\right)\cdot p^{\left(t-1\right)}\left(w_{ir}\;|\;w_{iq},c_{k}\right)\cdot p^{\left(t-1\right)}\left(w_{is}\;|\;w_{iq},w_{ir},c_{k}\right)\right]+\gamma_{2}\left[p^{\left(t-1\right)}\left(w_{iq}\;|\;c_{k}\right)\cdot p^{\left(t-1\right)}\left(w_{is}\;|\;w_{iq},c_{k}\right)\cdot p^{\left(t-1\right)}\left(w_{ir}\;|\;w_{iq},w_{is},c_{k}\right)\right]+\gamma_{3}\left[p^{\left(t-1\right)}\left(w_{ir}\;|\;c_{k}\right)\cdot p^{\left(t-1\right)}\left(w_{iq}\;|\;w_{ir},c_{k}\right)\cdot p^{\left(t-1\right)}\left(w_{is}\;|\;w_{iq},w_{ir},c_{k}\right)\right]+\gamma_{4}\left[p^{\left(t-1\right)}\left(w_{ir}\;|\;c_{k}\right)\cdot p^{\left(t-1\right)}\left(w_{is}\;|\;w_{ir},c_{k}\right)\cdot p^{\left(t-1\right)}\left(w_{iq}\;|\;w_{ir},w_{is},c_{k}\right)\right]+\gamma_{5}\left[p^{\left(t-1\right)}\left(w_{is}\;|\;c_{k}\right)\cdot p^{\left(t-1\right)}\left(w_{iq}\;|\;w_{is},c_{k}\right)\cdot p^{\left(t-1\right)}\left(w_{ir}\;|\;w_{iq},w_{is},c_{k}\right)\right]+\gamma_{6}\left[p^{\left(t-1\right)}\left(w_{is}\;|\;c_{k}\right)\cdot p^{\left(t-1\right)}\left(w_{ir}\;|\;w_{is},c_{k}\right)\cdot p^{\left(t-1\right)}\left(w_{iq}\;|\;w_{ir},w_{is},c_{k}\right)\right], \end{array}$$

(21) $$\begin{array}{c} p^{\left(t\right)}\left(y_{i}=c_{k}\;|\;x_{i}\right)=\frac{p^{\left(t-1\right)}\left(c_{k}\right)\cdot p^{\left(t-1\right)}\left(x_{i}\;|\;c_{k}\right)}{\sum_{k=1}^{\left|C\right|}p^{\left(t-1\right)}\left(c_{k}\right)\cdot p^{\left(t-1\right)}\left(x_{i}\;|\;c_{k}\right)}. \end{array}$$

For the M-step, the Laplace smoothing factor *λ* > 0 is implemented as well as in NB model to avoid zero count issue. However, with the BN dependency representation, there are four parameter estimation of the (*t* + 1)th iteration probability of *p*^(*t* + 1)^(*w*_*q*_ | *w*_*r*_, *w*_*s*_, *c*_*k*_), *p*^(*t* + 1)^(*w*_*q*_ | *w*_*r*_, *c*_*k*_), *p*^(*t* + 1)^(*w*_*q*_ | *c*_*k*_), and *p*^(*t* + 1)^(*c*_*k*_), which can be estimated from *t*th-iteration probability as expressed in

(22) $$\begin{array}{c} \begin{array}{l} p^{\left(t+1\right)}\left(w_{q}\;|\;c_{k}\right)=\frac{\lambda +\sum_{i=1}^{\left|X\right|}N\left(w_{q},x_{i}\right)p^{\left(t\right)}\left(y_{i}=c_{k}\;|\;x_{i}\right)}{\lambda \left|W\right|+\sum_{z=1}^{\left|W\right|}\sum_{i=1}^{\left|X\right|}N\left(w_{z},x_{i}\right)p^{\left(t\right)}\left(y_{i}=c_{k}\;|\;x_{i}\right)}, \end{array} \end{array}$$

(23) $$\begin{array}{c} \begin{array}{l} p^{\left(t+1\right)}\left(w_{q}\;|\;w_{r},c_{k}\right)=\frac{\lambda +\sum_{i=1}^{\left|X\right|}N\left(w_{q},x_{i}\right)p^{\left(t\right)}\left(y_{i}=c_{k}\;|\;w_{r},x_{i}\right)}{\lambda \left|W\right|+\sum_{z=1}^{\left|W\right|}\sum_{i=1}^{\left|X\right|}N\left(w_{z},x_{i}\right)p^{\left(t\right)}\left(y_{i}=c_{k}\;|\;w_{r},x_{i}\right)}, \end{array} \end{array}$$

(24) $$\begin{array}{c} \begin{array}{l} p^{\left(t+1\right)}\left(w_{q}\;|\;w_{r},w_{s},c_{k}\right)=\frac{\lambda +\sum_{i=1}^{\left|X\right|}N\left(w_{q},x_{i}\right)p^{\left(t\right)}\left(y_{i}=c_{k}\;|\;w_{r},w_{s},x_{i}\right)}{\lambda \left|W\right|+\sum_{z=1}^{\left|W\right|}\sum_{i=1}^{\left|X\right|}N\left(w_{z},x_{i}\right)p^{\left(t\right)}\left(y_{i}=c_{k}\;|\;w_{r},w_{s},x_{i}\right)}, \end{array} \end{array}$$

whereas *W* is a total number of terms and any term *w*_*z*_ ∈ *W*.

(25) $$\begin{array}{c} p^{\left(t+1\right)}\left(c_{k}\right)=\frac{\lambda +\sum_{i=1}^{\left|X\right|}p^{\left(t\right)}\left(y_{i}=c_{k}\;|\;x_{i}\right)}{\lambda \left|C\right|+\left|X\right|}. \end{array}$$

Then, we can derive *p*^(*t* + 1)^(*w*_*r*_ | *c*_*k*_) and *p*^(*t* + 1)^(*w*_*s*_ | *c*_*k*_) using the similar calculation as (22). For the dependency representations of two random variables *w*, that is, *p*^(*t* + 1)^(*w*_*q*_ | *w*_*s*_, *c*_*k*_), *p*^(*t* + 1)^(*w*_*r*_ | *w*_*q*_, *c*_*k*_), *p*^(*t* + 1)^(*w*_*r*_ | *w*_*s*_, *c*_*k*_), *p*^(*t* + 1)^(*w*_*s*_ | *w*_*q*_, *c*_*k*_), and *p*^(*t* + 1)^(*w*_*s*_ | *w*_*r*_, *c*_*k*_) can be computed by following the similar approach as (23). Similarly, the estimation of *p*^(*t* + 1)^(*w*_*r*_ | *w*_*q*_, *w*_*s*_, *c*_*k*_) and *p*^(*t* + 1)^(*w*_*s*_ | *w*_*q*_, *w*_*r*_, *c*_*k*_) can be obtained by the same way as shown in (24). Finally, the coefficients *γ*, *β*, and *α* of interpolation approach are employed in order to weigh the knowledge from multiple dependency representations.  Algorithm 1 explains pseudocode for iEM model, and Algorithm 2 expresses our proposed dEM method.

### 3.5. The Incorporation of Unlabeled Data

In the environment of insufficient labeled data, SSL is one solution that utilizes an inexpensive and ubiquitous source of data. The transductive learning [65], one type of SSL, begins its process with making use of a limited number of labeled data (*𝒟*_L_) to build a rough model and then aggregated a large number of unlabeled data (*𝒟*_U_) (test set) to revise and improve the model iteratively. In the experiment, we investigated the three alternative approaches of initialization and iterative weighting of relation labels for unlabeled data incorporation.

- *T*_*p*_*M*_*L*___: This method is equivalent to the general transductive learning, in which the label of the test set *𝒟*_U_ can be derived by a classifier that is trained on the *𝒟*_L_. Then, the augmented *𝒟*_L_ with the labeled *𝒟*_U_, so called *𝒟*_L+U_, is used for the further iteration.
- *T*_*p*_0.5__: The class probability of the *𝒟*_U_ is equally assigned to *𝒟*_L_ and used as an initial probability. In this approach, the *𝒟*_L+U_ can be derived earlier and integrated in training process for the first iteration. Therefore, in the next iteration, the *𝒟*_U_ is not strictly guided by the labeled data. The revision process is probably the same manner to the previous method by combining both data set *𝒟*_L+U_ for the further iteration.
- *T*_*p*_random__: Similarly, the initial probability of *𝒟*_U_ is assigned randomly rather than the fixed value of 0.5. The degree of likelihood for each label can be varied from 0 to 1 whereas the total probability of ADR and IND labels equals 1.

In order to evaluate our proposed method, three types of text representation across three parameters of unlabeled data incorporation are investigated. Finally, our proposed methods and its enhancement, MIL-dEM-S-S (supervised learning) and MIL-dEM-T-S methods (transductive learning), are compared to TSVM and three MIL models, MISVM, MINB, and MILR, which are implemented in WEKA [66].

## 4. Evaluation

We assess our proposed method using various parameter settings as shown in Table 3 and evaluate by the *hold-out evaluation* through the *k*-fold cross validation whereas *k* = 5. The three main measures as defined by (26), (27), and (28), that is, precision, recall, and F1, are used for model evaluation, while the positive class in our experiments is ADR label. In our experiment, we use MetaMap Java API for NER and Stanford CoreNLP Java API for OpenIE and implement Python program for EM-based methods. For model comparison, we execute WEKA Java-based software and SVM*^light^* (http://svmlight.joachims.org), which is implemented in C programming language, on Mac OS with Intel Core i5 processor running at 2.5 GHz and 8 GB of physical memory.

(26) $$\begin{array}{c} precision=\frac{tp}{tp+fp}, \end{array}$$

(27) $$\begin{array}{c} recall=\frac{tp}{tp+fn}, \end{array}$$

(28) $$\begin{array}{c} F1=\frac{2\times precision\times recall}{precision+recall}. \end{array}$$

### 4.1. Data

Our proposed framework is examined on the unstructured texts from EMRs of intensive care unit which is derived from MIMIC-III [67]. The data is freely available at *PhysioNet* (https://mimic.physionet.org) and is accessed on Apr 25, 2016 with the version 1.3. The over 58,000 hospital admissions for 38,645 adults and 7875 neonates are presented in the data source spanning up to 12 years from June 2001. In our work, the discharge summary from two main hospital sections, that is, brief hospital course (BHC) and the history of present illness (HPI) are preliminary explored. For data preparation, we employ SBD, stop word removal, tokenization, NER, and normalization. We consider two semantic types of UMLS CUI regarding CHEM and DISO for drug and event entities, respectively. As the results, nearly 1.6 million sentences are extracted and used as our corpus.

### 4.2. Results and Discussion

We conduct four main experiments in order to evaluate the effectiveness of our proposed method: (i) the key phrasal pattern analysis, (ii) the evaluation on the effectiveness of the key phrasal patterns, (iii) the effectiveness of the pattern-based feature with MIL-iEM and MIL-dEM, and (iv) the evaluation on overall performance with advanced machine learning methods.

#### 4.2.1. Key Phrasal Pattern Analysis

We initially analyze the discovered key phrasal patterns to investigate the degree of characterization of relation labels. Given a key phrasal pattern *pattern*, we compute the pattern score (*S*) by performing the conditional entropy (*H*) inversion and polarity adjustment to visualize the performance of the extracted key phrasal patterns.

(29) $$\begin{array}{c} H=-p\left(ADR\;|\;\;pattern\right)\log_{2}\;p\left(ADR\;|\;\;pattern\right)-p\left(IND\;|\;\;pattern\right)\log_{2}\;p\left(IND\;|\;\;pattern\right)S=SIGN\left(0.5-p\left(IND\;|\;\;pattern\right)\right)\times \left(1-H\right). \end{array}$$

**Figure 5:**
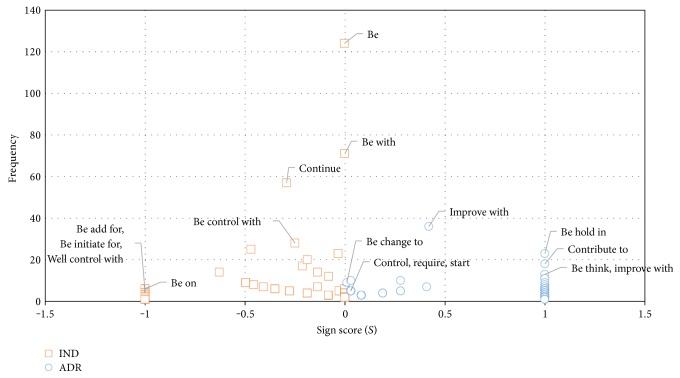
The *x-*axis exhibits the pattern score with polarity whereas the score > 0 represents the distribution of pattern relevant to ADR (blue circle marker), the score < 0 represents the distribution of pattern relevant to IND (orange square marker), and score = 0 indicates no relevance between pattern and both labels. The *y-*axis is the frequency of patterns that appear in the clinical texts.

From Figure 5, a pattern that is located far from the middle line (score 0) and closed to the top left or the top right corners expresses the high effectiveness of semantic discrimination ability relevant relation labels. For example, the key phrasal patterns “be-hold-in,” “contribute-to,” “be-think,” and “improve-with” are strongly relevant to ADR label and “be-add-for,” “be-initial-for,” and “be-on” are rather associated to IND. Opposite to the key phrasal patterns, “be” and “be-with” are presented near the middle line in the graph that indicates the fuzziest patterns.

Additionally, the figure clearly illustrates that the patterns relevant to ADR are more efficient than the pattern relevant to IND, the small number of ADR patterns are located nearby the original point, and most of the ADR patterns are placed with spread distance. On the one hand, patterns relevant to IND are presented to dense at the location which is nearly zero score and zero frequency.  Table 4 presents the example of the sentences that are relevant to key phrasal patterns and pattern direction. Finally, the key phrasal patterns with a pattern score over than the threshold are selected for the further process.

#### 4.2.2. Evaluation on the Effectiveness of the Pattern-Based Feature

The comparison of the multiple feature types across varying of initial weighting of relation labels for unlabeled data incorporation throughout the MIL-iEM are assessed in order to examine the effectiveness of the pattern-based feature. We divide the experiments into two parts based on the decision methods in EM algorithm. The former refers to *soft decision making* (MIL-iEM-S) in which the predicted result is directly yielded by the estimated class probability. The latter is so-called *hard decision making* (MIL-iEM-H) in which the predicted outcome is considered the cutoff value of the probability and assigned class label instead of likelihood ratio. We initially perform the experimental setting on the traditional-independent assumption through MIL-iEM model.

Table 5 expresses an assessment of five text transformation across three alternative document representations and three initial weighting of unlabeled data *𝒟*_U_ based on *soft decision making* and *hard decision making*. In the table, the pattern-based feature is expressed in the top 4 of each experimental setting, that is, *S*–*P*, *S*–*W*, *L*–*P*, and *L*–*W*. From the experimental results, we found that the pattern-based feature outperformed traditional bag-of-words (BOW). The highest *F*1 score value, 0.841, is resulted by MIL-iEM-SP-TF-S-T_*p*_0.5__ model which outperformed the baseline MIL-iEM-BOW-TF-S-T_*p*_0.5__ up to 4.4%. In addition, *B* and TF document representations have slightly better performance than TFIDF for all types of initial weighting method. The similar results are found on *hard decision making* approach as well. The pattern-based feature performed better performance than *BOW* feature. The MIL-iEM-LW-TFIDF-H-T_*p*_0.5__ model obtains the highest performance of F1 score 0.807 and 3.3% improvement from the MIL-iEM-BOW-TFIDF-H-T_*p*_0.5__ baseline model. However, it is noticed that the *hard decision making* results in poor performance when compared to the soft version.

**Figure 6:**
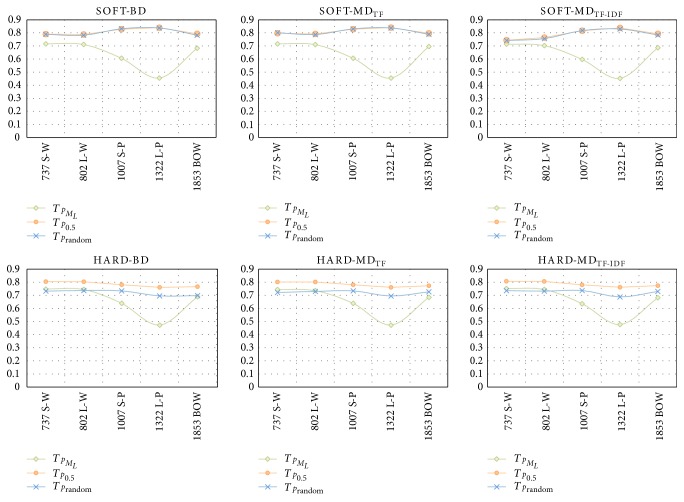
The number of features for each type of feature extraction and weighting method across F1 score. (a) represents the *soft decision* method and (b) represents the *hard decision* method of MIL-iEM.

The performance comparison across the number of features is exhibited in Figure 6. The number of features relevant to pattern-based features is ranged from 737 to 1322 dimensions, and the number of BOW feature is 1853 dimensions. From the graph, even though our proposed pattern-based features with MIL-iEM-T_*p*_0.5__ and MIL-iEM-T_*p*_random__ provide slightly different F1 score from the BOW feature, their number of dimension are less than half of BOW, especially *S–W* and *L–W* features. Therefore, our proposed pattern-based feature is more efficient than BOW feature due to the small number of features but yield similar model performance.

Accordingly, the experimental results confidently support that the simplified sentence using relation tuple of a drug, a key phrasal pattern, and an event is a potential feature transformation for relation classification task. Moreover, ignoring the insignificant contexts can reduce redundancy of feature and avoid computational time issue that is frequently caused by the curse of dimensions.

#### 4.2.3. Evaluation on the Effectiveness of MIL-dEM-SL and MIL-dEM-T

In this experiment, the comparison between our proposed method based on SL (MIL-dEM-SL) and transductive learning (MIL-dEM-T) across varying parameters such as feature types, pattern-weighting models, and the initial weight methods for unlabeled data incorporation are examined. Our proposed method is based on dependency representation of texts, and the posterior estimation is based on the interpolation of Markov property. The experiment is set up with supervised learning-based model and three transductive learning-based models with different initial weight methods of *𝒟*_U_ incorporation. The two types of pattern-based features such as surface lexicon-based (*S–P*) and syntactically lemmatized lexicon-based (*L–P*) are used for examination. The parameter tuning is also performed for all approaches.

As the results in Table 6, among transductive learning models, the performance of *S–P* feature is slightly different from *L–P* feature for all models. The simple binary (*B*) weighting model presents the higher F1 score over TF and TFIDF. Moreover, MIL-dEM-S-T_*p*_*M*_*L*___ model exhibits the higher performance than the fuzzy guideline by MIL-dEM-S-T_*p*_0.5__ and MIL-dEM-S-T_*p*_random__ models for all evaluation matrices.

On the other hand, the F1 score of MIL-dEM-SP-S-SL surface lexicon-based feature is better than MIL-dEM-LP-S-SL syntactically lemmatized lexicon-based feature with 1% and 0.8% for TF- and TFIDF-weighting model, respectively.

Similarly, the F1 score of the pattern-based feature *S–P* across the three types of pattern-weighting model, that is, *B*, TF, and TFIDF models is also slightly different; 0.928 for MIL-dEM-SP-B-S-SL, 0.946 for MIL-dEM-SP-TF-S-SL, and 0.938 for MIL-dEM-SP-TFIDF-S-SL. Among models within MIL-dEM-S-SL setting, the highest F1 score is presented by TF-weighting model with 0.946.

One of the interesting results shows that the unlabeled data incorporation is exhibited to increase the model performance. The highest effectiveness, 0.954 of F1 score, is presented by MIL-dEM-SP-B-S-T_*p*_*M*_*L*___ model which is the simple binary weighting model, and the model shows 2.6%, 1.6%, and 0.8% improvement over MIL-dEM-SP-B-S-SL, MIL-dEM-SP-TFIDF-S-SL, and MIL-dEM-SP-TF-S-SL, the best performance of our proposed supervised learning, respectively.

According to the result from the parameter optimization of our proposed method, the model performance is strongly relevant to the dependency representation of random variables as follows: (i) an event and the clinical outcome and (ii) a pattern, a drug, and the clinical outcome. In the contrast, the model is shown to have less relevance between a drug and an event or a pattern and an event.

#### 4.2.4. Evaluation on Overall Performance with Advanced Machine Learning Methods

The comparison of our proposed method and advanced machine learning methods is presented in Table 7. The best models of each set of models are used for assessment. The well-known MIL methods, that is, MISVM, MINB, MILR are executed using WEKA. On the one hand, we customize the original TSVM using the source code from the author and incorporate the MIL assumption as discussed in the previous section (see Section 2.2). We divide the discussion into three parts: the effectiveness of supervised learning model, the effectiveness of transductive learning model, and the overall performance.

Firstly, the experimental results among baseline-supervised learning methods, that is, MISVM-TFIDF, MINB-B, and MILR-B, show that BOW feature works well for all MIL methods; conversely, the pattern-based feature *S–P* contributes a dramatic improvement when combined with our proposed method MIL-dEM-TF-S-SL. The TFIDF*-*weighting model yields the high performance for MISVM with F1 score 0.901, while binary weighting model (*B*) is exhibited to improve the performance for MINB and MILR with F1 scores 0.880 and 0.861, respectively. However, our proposed MIL-dEM-TF-S-SL with *S–P* feature outperforms all MIL methods, and 4.5% F1 score is better than the highest performance of advanced machine learning method which is resulted by MISVM-TFIDF with BOW feature. The precision of MIL-dEM-TF-S-SL with *S–P* feature is slightly lower than MISVM-TFIDF with BOW but the recall is significantly improved. Accordingly, our proposed method contributes to reducing the type II error which is always considered in the medical domain.

Secondly, the comparison among transductive learning methods, the BOW feature with TSVM-B is shown to achieve an F1 score of 0.889, while applying the pattern-based feature *S–P*, its performance is presented to degrade around 2%. Conversely, the pattern-based feature *S–P* with MIL generative method exhibits to enhance the effectiveness of the models. The accuracy of MIL-iEM-TF-S-T_*p*_0.5__ model increases up to 6.3% when the pattern-based feature is deployed instead of the BOW feature.

Lastly, in the overall evaluation, the generative models with dependency representation, that is, MIL-dEM-TF-S-SL and MIL-dEM-B-S-T_*p*_*M*_*L*___, outperform for all models. The highest performance is exhibited by our transductive learning MIL-dEM-B-S-T_*p*_*M*_*L*___ method with 0.934 precision, 0.975 recall, 0.954 F1 score, and 0.949 accuracy, respectively. Moreover, improving the generative model by substitute assumption of word-dependency MIL-dEM-B-S-T_*p*_*M*_*L*___ model to word-independency MIL-iEM-TF-S-T_*p*_0.5__ model is shown to dramatically improve 11.3% F1 score and 12.2% accuracy.

From multiple aspect assessments, the experimental results confidently support that our proposed method, MIL with the two generative models, has the comparative advantage in performance for relation classification task. The proposed pattern-based feature contributes to reduce the curse of dimension issue and preserve text dependency structure. The incorporation of a generative model with proper model assumption and transductive learning can potentially estimate the distribution of patterns relevant to harmful or beneficial event of drug usage with high precision and recall. Our proposed method can provide the supporting evidence based on the relevant clinical sentence rather than only prediction of result which is expected to further assist a professional medical for decision making on treatment or diagnosis process.

## 5. Conclusion

This paper presents a framework of distant supervision with MIL and transductive learning for detecting adverse reaction hidden in clinical texts. Our work aims to deal with two main difficulties: (i) the limitation of hand-labeled data and (ii) intractable processing of large-scale unstructured clinical texts.

The first issue is coped with distant supervision paradigm by knowledge base incorporation. Therefore, we can automatically assign either ADR or IND label to each drug-event pair and use as labeled examples. For the second issue, we propose the pattern-based feature to present semantic comprehension of a sentence and proposed alternative parameters learning of a generative model using dependency representation model assumption. However, such training data set derived by distant supervision is formed as the instance-level, while the predictive goal is focused on the entity-level. Therefore, MIL paradigm is involved into the framework. The collected statistics from the tagged drug-event pairs are used to examine the semantic distribution relevant to ADR and IND. Exploiting EM algorithm as the base model for our supervised learning and transductive learning, it is helpful to estimate the probability of an unknown relation of given drug-event pair and then classify this relation to either ADR or IND. From the experimental results on multiple assessments, we found three significant findings.

Firstly, the pattern-based feature contributes to improve model performance of generative models. The MIL-iEM-SP-TF-S-T_*p*_0.5__ model is shown to achieve the highest performance among all MIL-iEM-based methods with 0.844 precision, 0.838 recall, and 0.841 F1 score, and the model provides the outstanding improvement over the traditional BOW method, MIL-iEM-BOW-TF-S-T_*p*_0.5__ model, up to 4.4% F1 score.

The second potential result, the traditional assumption of word independency is rather improper for natural clinical texts. Therefore, we tackle such fundamental problem by integrating Markov assumption on dependency representation of texts in order to estimate the prior probability and likelihood probability in a generative model. Given the same set of the pattern-based input features, the performance of MIL-dEM model is dramatically improved from MIL-iEM model. The MIL-dEM-SP-B-S-T_*p*_*M*_*L*___ model exhibits the improvement over MIL-iEM-SP-B-S-T_*p*_0.5__ up to 8.9% precision, 13.9% recall, and 11.4% F1 score.

Lastly, the incorporation of unlabeled data *𝒟*_U_ and labeled one *𝒟*_L_ using MIL-dEM-SP-B-S-T_*p*_*M*_*L*___ model achieves the highest effectiveness with 0.954 F1 score. In addition, our proposed MIL-dEM-SP-B-S-T_*p*_*M*_*L*___ model also outperforms the advanced machine learning methods by F1 score improvement up to 5.3% of MISVM-BOW-TFIDF, 7.4% of MINB-BOW-B, 9.3% of MILR-BOW-B, 6.5% of TSVM-BOW-B, and 11.3% of MIL-iEM-SP-TF-S-T_*p*_0.5__.

However, our work presents some limitations that can contribute to support further improvement of the framework. The projection from distant supervision to corpus currently is employed by MetaMap tools and can be improved by advance method such as word embedding to increase high potential entity-level relation for instance examples. The key phrasal pattern extraction in the current work is scoped by the sentence boundary, but a drug and an event possibly associate throughout across different sentences. This issue would be challenged by coreference problem. Even though the discovered key phrasal patterns provides the significant role for relation classification, the number of patterns is rather limited and probably encounters the problem of out of vocabulary (OOV) when applied to the framework with a huge unseen data. Therefore, the semantic representation is the promising method to increase the number of key phrasal patterns.

## Figures and Tables

**Algorithm 1:**
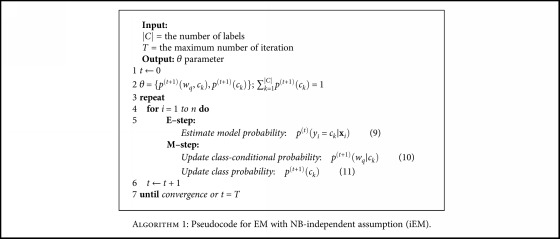
Pseudocode for EM with NB-independent assumption (iEM).

**Algorithm 2:**
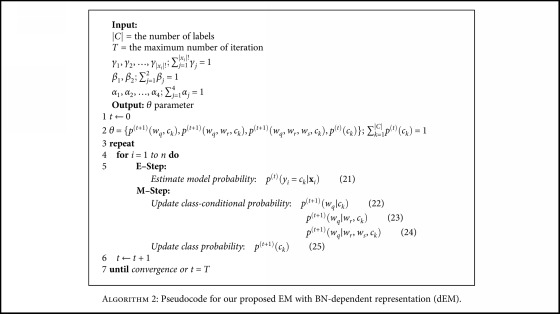
Pseudocode for our proposed EM with BN-dependent representation (dEM).

**Table 1:** A list of previous studies on ADR identification from unstructured text.

Data source	Literature	Year	Size	Label number	Labeling method	NER	Method
*Supervised learning*							
EMR	Aramaki et al. [10]	2010	3012 notes	A, O (2)	H	CRF	Pattern-based
	Sohn et al. [11]	2011	237 notes	A, O (2)	H	cTAKES	Pattern-based, DT C4.5
	Henriksson et al. [26]	2015	400 notes	A, I, O (3)	H	CRF	Word embedding, RF
	Casillas et al. [12]	2016	n/a	A, O (2)	H	FreeLing-Med	Pattern-based, SVM, RF
Literature	Peng et al. [16]	2016	18,410 abstracts	A, O (2)	H, DS	Dictionary, tmChem, DNorm	Feature-based, SVM
Social media	Segura-Bedmar et al. [33]	2015	84,000 messages	A, I (2)	DS	GATE	Shallow linguistic kernel, distant supervision
	Nikfarjam et al. [17]	2015	8800 blog sentences, 3200 tweets	A, I, O (3)	H	CRF	Word embedding, CRF
	Jenhani et al. [18]	2016	80,000 tweets	A, O (2)	R, ODIN	Dictionary, Stanford CoreNLP	Rule-base, feature-based, DT, SVM, LR, NB
	Liu et al. [34]	2016	1800 blog sentences, 500 tweets	A, O (2)	H	MetaMap	Feature-based, tree kernel-based, ensemble method

*Semisupervised learning*							
EMR	Taewijit and Theeramunkong [13]	2016	1.5 M sentences	A, I (2)	DS	MetaMap	Distant supervision, OpenIE [35], pattern-based
Literature	Kang et al. [36]	2014	1644 abstracts	A, O (2)	H	Peregrine	Hierarchical graph-based, shortest path
Social media	Liu and Chen [37]	2015	400 sentences	A, I, O (3)	H	MetaMap	Dependency tree, TSVM [38]

*Unsupervised learning*							
EMR	Wang et al. [39]	2009	25,074 notes	None	None	MedLEE	Co-occurrence
Literature	Xu and Wang [14]	2014	119 M sentences	None	None	Parse tree	Pattern-based, ranking
Social media	Feldman et al. [15]	2015	0.1~1 M messages	None	None	Dictionary, pattern	HPSG-based parser, postprocessing of relation merging

Labels: A = ADR; I = IND; O = other (ADR cause, ADR outcome, non-ADR, negated ADR, others); labeling method: DS = distant supervision, H = human; R = rule-based.

**Table 2:** Types of feature extraction for a given sentence. Here, the first character is either *L* (syntactically lemmatized lexicon) or *S* (surface lexicon), and the second character is either *P* (phrase) or *W* (word). BOW stands for bag-of-words. CUI C0033487 is a UMLS concept of propofol. C0031469 is a UMLS concept of phenylephrine. CUI C0020649 is a UMLS concept of hypotension.

Sentences	Types	Example of feature representation	Example of drug-event candidates (*d*, *p*, *e*, *y*)
On arrival here, propofol was held due to hypotension.	*L–P*	C0033487 be-hold-due-to C0020649	(C0033487, be-hold-due-to, C0020649, ADR)
	*L–W*	C0033487 be hold due to C0020649	NA
	*S–P*	C0033487 was-held-due-to C0020649	(C0033487, was-held-due-to, C0020649, ADR)
	*S–W*	C0033487 was held due to C0020649	NA
	BOW	On arrival here, propofol was held due to hypotension.	NA

Phenylephrine drip was started for hypotension.	*L–P*	C0031469 be-start-for C0020649	(C0031469, be-start-for, C0020649, IND)
	*L–W*	C0031469 be start for C0020649	NA
	*S–P*	C0031469 was-started-for C0020649	(C0031469, was-started-for, C0020649, IND)
	*S–W*	C0031469 was started for C0020649	NA
	BOW	Phenylephrine drip was started for hypotension.	NA

**Table 3:** The list of parameters for assessment.

Parameter group	Parameter type	Parameter subtype	Parameter name	Variable name
Document representation		Syntactic lemmatization	Syntactically lemmatized lexicon	*L*
			Surface lexicon	*S*
		Pattern granularity	Phrase form	*P*
			Word form	*W*
	Pattern-weighting models	Bernoulli	Binary	*B*
		Multinomial	TF (term frequency)	TF
			TFIDF (TF-inverse document frequency)	TFIDF

Model assumption	Independent assumption	EM with naïve Bayes		iEM
	Dependency representation assumption	EM with Bayesian network		dEM

Model decision method	Soft decision making			*S*
	Hard decision making			*H*

Learning method	Supervised learning			SL
	Transductive learning	Initial weight method for unlabeled data	Supervised model	*T*_*p*_*M*_*L*___
			Equal probability	*T*_*p*_0.5__
			Random probability	*T*_*p*_random__

**Table 4:** Example of relevant sentences of pairs of drug-event (*d*, *p*, *e*).

Drugs (d)	Key phrasal patterns (p)	Events (e)	Pattern direction	Sentences
*ADR*				
C0020261 (hydrochlorothiazide)	be-hold-in	C0020625 (hyponatremia)	d → e	However the patient's sodium was 131 on discharge thus the patient's HCTZ was-held-in the setting of hyponatremia.
C0000970 (acetaminophen)	be-think	C0002871 (anemia)	e → d	Her anemia is-thought to be due to direct effects of acetaminophen on marrow or indirect via kidneys.

*IND*				
C0020223 (hydrallazine)	be-give-for	C0020538 (hypertension)	d → e	Hydrallazine 20 mg IV was-given-for isolated episode of hypertension and emesis ensued.
C0043031 (warfarin)	be-initiate-for	C0004238 (atrial fibrillation)	e → d	Warfarin was-initiated-for his atrial fibrillation with an initial heparin bridge.

Pattern direction: d → e is drug-event; e → d is event-drug.

**Table 5:** The effectiveness comparison on fivefold cross-validation of text transformation across three types of document representation using MIL-iEM with *soft decision making* (MIL-iEM-S) and *hard decision making* (MIL-iEM-H).

Models	Soft decision making									Hard decision making								
	B			TF			TFIDF			B			TF			TFIDF		
	*P*	*R*	F1	*P*	*R*	F1	*P*	*R*	F1	*P*	*R*	F1	*P*	*R*	F1	*P*	*R*	F1
	MIL-iEM-S-T_*p*_*M*_*L*___									MIL-iEM-H-T_*p*_*M*_*L*___								
*S–P*	0.858	0.308	0.454	0.858	0.308	0.454	0.857	0.307	0.452	0.856	0.327	0.473	0.856	0.327	0.473	0.858	0.330	0.477
*S–W*	0.879	0.599	0.712	0.873	0.600	0.711	0.871	0.589	0.703	0.871	0.651	0.745	0.863	0.642	0.736	0.873	0.650	0.745
*L–P*	**0.890**	0.460	0.606	**0.890**	0.460	0.606	**0.882**	0.451	0.597	**0.887**	0.500	0.639	**0.887**	0.500	0.639	**0.881**	0.498	0.636
*L–W*	0.868	0.609	**0.716**	0.863	0.611	**0.716**	0.873	0.604	**0.714**	0.866	**0.659**	**0.748**	0.863	**0.653**	**0.744**	0.868	**0.662**	**0.752**
*BOW*	0.755	**0.624**	0.683	0.780	**0.628**	0.696	0.765	**0.624**	0.687	0.744	0.642	0.690	0.730	0.646	0.685	0.726	0.642	0.682
	MIL-iEM-S-T_*p*_0.5__									MIL-iEM-H-T_*p*_0.5__								
*S–P*	**0.845**	0.836	**0.840**	**0.844**	0.838	**0.841**	**0.846**	0.830	**0.838**	0.620	**0.985**	0.761	0.620	**0.985**	0.761	0.621	**0.985**	0.762
*S–W*	0.783	0.792	0.788	0.784	0.801	0.792	0.787	0.743	0.764	0.686	0.971	0.804	0.683	0.969	0.802	0.691	0.967	0.806
*L–P*	0.840	0.816	0.828	0.840	0.816	0.828	0.836	0.799	0.817	0.651	0.974	0.781	0.652	0.974	0.781	0.651	0.973	0.780
*L–W*	0.785	0.797	0.791	0.796	0.799	0.798	0.777	0.714	0.744	**0.693**	0.962	**0.805**	**0.689**	0.960	**0.802**	**0.696**	0.960	**0.807**
BOW	0.692	**0.927**	0.793	0.749	**0.850**	0.797	0.735	**0.861**	0.793	0.632	0.973	0.766	0.646	0.962	0.773	0.649	0.960	0.774
	MIL-iEM-S-T_*p*_random__									MIL-iEM-H-T_*p*_random__								
*S–P*	**0.840**	0.836	**0.838**	**0.842**	**0.834**	**0.838**	**0.841**	0.819	**0.830**	0.645	0.755	0.696	0.644	0.754	0.695	0.630	0.757	0.688
*S–W*	0.773	0.790	0.782	0.782	0.792	0.787	0.782	0.732	0.756	0.666	0.825	**0.737**	0.649	**0.834**	0.730	0.640	0.856	0.732
*L–P*	0.833	0.830	0.832	0.833	0.828	0.831	0.835	0.801	0.818	**0.668**	0.814	0.734	**0.668**	0.814	**0.734**	0.656	0.841	**0.737**
*L–W*	0.783	0.796	0.789	0.799	0.805	0.802	0.778	0.710	0.742	0.657	**0.827**	0.732	0.642	0.823	0.722	0.641	**0.863**	0.736
BOW	0.691	**0.900**	0.782	0.748	**0.834**	0.789	0.734	**0.841**	0.784	0.655	0.746	0.698	0.666	0.801	0.727	**0.675**	0.794	0.730

B: binary frequency; TF: term frequency; TFIDF: term frequency-inverse document frequency.

**Table 6:** The effectiveness of MIL-dEM-S-SL and MIL-dEM-S-T comparison across three types of initial weight on fivefold cross-validation with *soft decision making*.

	Models	B			TF			TFIDF		
		*P*	*R*	F1	*P*	*R*	F1	*P*	*R*	F1
Supervised learning	MIL-dEM-S-SL^1^									
	*S–P*	0.883	**0.978**	**0.928**	**0.904**	**0.993**	**0.946**	**0.890**	**0.993**	**0.938**
	*L–P*	**0.896**	0.962	**0.928**	0.898	0.978	0.936	0.889	0.976	0.930

Transductive learning	MIL-dEM-S-T_*p*_*M*_*L*___^2^									
	*S–P*	**0.934**	**0.975**	**0.954**	0.901	**0.942**	**0.921**	**0.881**	**0.951**	**0.915**
	*L–P*	0.926	0.962	0.944	**0.919**	0.916	0.918	0.875	0.945	0.909
	MIL-dEM-S-T_*p*_0.5__^3^									
	*S–P*	0.839	**0.907**	**0.872**	0.635	**0.925**	0.754	0.686	**0.916**	0.784
	*L–P*	**0.850**	0.889	0.869	**0.663**	0.900	**0.763**	**0.714**	0.887	**0.791**
	MIL-dEM-S-T_*p*_random__^4^									
	*S–P*	0.830	**0.889**	**0.859**	0.581	0.607	0.594	0.647	**0.682**	0.664
	*L–P*	**0.843**	0.865	0.854	**0.597**	**0.619**	**0.608**	**0.657**	0.679	**0.668**

^1,2^ *γ* = [0.45 0.02 0.45 0.02 0.04 0.02], *β* = [0.97 0.02 0.01 0.00], *α* = [0.10 0.90]; ^3,4^*γ* = [0.45 0.02 0.45 0.02 0.04 0.02], *β* = [1.00 0.00 0.00 0.00], *α* = [0.50 0.50]. B: binary frequency; TF: term frequency; TFIDF: term frequency-inverse document frequency.

**Table 7:** The comparison of overall performance among MIL-dEM-SL, MIL-dEM-T, advanced machine learning methods, and MIL-iEM-T using fivefold cross-validation.

Models	BOW				*S–P*			
	*P*	*R*	F1	Acc.	*P*	*R*	F1	Acc.
*Supervised learning*								
MIL-dEM-TF-S-SL^1^	—	—	—	—	**0.904**	**0.993**	**0.946**	**0.939**
MISVM-TFIDF^2^	**0.918**	0.885	**0.901**	**0.895**	0.799	0.733	0.765	0.735
MINB-B	0.864	**0.896**	0.880	0.867	0.619	0.701	0.744	0.691
MILR-B^3^	0.869	0.852	0.861	0.850	0.718	0.783	0.749	0.692

*Transductive learning*								
MIL-dEM-B-S-T_*p*_*M*_*L*___^4^	—	—	—	—	**0.934**	**0.975**	**0.954**	**0.949**
TSVM-B	**0.898**	**0.881**	**0.889**	0.881	0.873	0.865	0.869	0.859
MIL-iEM-TF-S-T_*p*_0.5__	0.749	0.850	0.797	0.764	0.844	0.838	0.841	0.827

^1,4^ *γ* = [0.45 0.02 0.45 0.02 0.04 0.02], *β* = [0.97 0.02 0.01 0.00], *α* = [0.10 0.90]. ^2^Polynomial kernel, *C = 10*. ^3^Collective MI assumption, geometric mean for posteriors.
