# Corrigendum to “Distant Supervision with Transductive Learning for Adverse Drug Reaction Identification from Electronic Medical Records”

**DOI:** 10.1155/2018/8731097

**Published:** 2018-05-02

**Authors:** Siriwon Taewijit, Thanaruk Theeramunkong, Mitsuru Ikeda

**Affiliations:** ^1^The School of Information, Communication and Computer Technologies, Sirindhorn International Institute of Technology, Thammasat University, Pathum Thani 12120, Thailand; ^2^The School of Knowledge Science, Japan Advanced Institute of Science and Technology, Nomi 923-1292, Japan

In the article titled “Distant Supervision with Transductive Learning for Adverse Drug Reaction Identification from Electronic Medical Records” [1], there was a missing grant number. Thus, the Acknowledgements section should be updated as follows:

The authors would like to express their gratitude to Dr. Sewan Theeramunkong (Pharmacy), Thammasat University; Dr. Ithipan Methasate, NECTEC; and Professor Dr. Kenji Araki (MD), University of Miyazaki Hospital for relation validation and very helpful suggestions. The authors would like to thank a workshop on Big Data Analytics-as-a-Service: Architecture, Algorithms, and Application in Health Informatics, 23rd ACM SIGKDD Conference on Knowledge Discovery and Data Mining (KDD2017), 2017 that provided a great opportunity for oral presentation and the anonymous reviewers for their valuable comments and suggestions. The work was supported by Thailand Research Fund under Grant no. RTA6080013 and the SIIT-JAIST-NECTEC Doctoral Dual Degree Program and National Research University project (NRU) and Intelligent Informatics and Service Innovation (IISI, SIIT), Center of Excellence in Intelligent Informatics, Speech and Language Technology and Service Innovation (CILS), Thammasat University, Thailand.
